# Increasing patient participation in oncology clinical trials

**DOI:** 10.1002/cam4.5150

**Published:** 2022-08-31

**Authors:** Jie Chen, Ying Lu, Shivaani Kummar

**Affiliations:** ^1^ Department of Biometrics Overland Pharmaceuticals Dover Delaware USA; ^2^ Department of Biomedical Data Science and Stanford Cancer Institute Stanford University Palo Alto California USA; ^3^ Knight Cancer Institute Oregon Health & Science University Portland Oregon USA

**Keywords:** cancer trials, clinical trials, patient accrual, pragmatic clinical trials

## Abstract

**Aim:**

Timely recruitment of eligible participants is essential for the success of clinical trials, with insufficient accrual being the leading cause for premature termination of both oncology and non‐oncology trials.

**Methods:**

In this paper we further elaborate on the challenges for patient participation in oncology trials from physician, patient, healthcare system, and some trial‐related perspectives.

**Results:**

We present strategies such as use of digital healthcare technologies, real‐world data and real‐world evidence, decentralized clinical trials, pragmatic trial designs, and supportive services to increase patient participation.

**Conclusions:**

Multifaceted measures are necessary to increase patient participation, especially for those who are under‐represented in cancer trials.

## Graphical abstract

Timely recruitment of eligible participants is essential for the success of clinical trials, with insufficient accrual being the leading cause for premature termination of both oncology and non‐oncology trials. In this paper we further elaborate on the challenges for patient participation in oncology trials from physician, patient, healthcare system, and some trial‐related perspectives and present strategies such as use of digital healthcare technologies, real‐world data and real‐world evidence, decentralized clinical trials, pragmatic trial designs, and supportive services to increase patient participation.

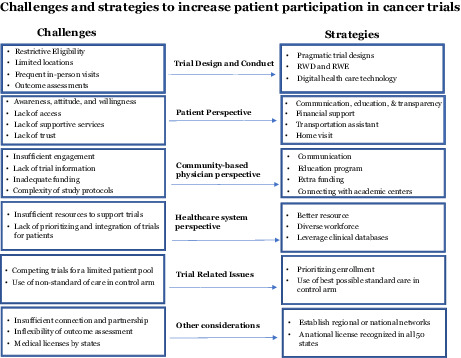

## 1 INTRODUCTION

Timely recruitment of eligible participants is essential for the success of clinical trials, with insufficient accrual being the leading cause for premature termination of both oncology and non‐oncology trials.^1^, ^2^, ^3^ A recent review found patient participation in oncology trials to be low, ranging from 2% to 8% of adult cancer patients.^4^ A meta‐analysis of studies conducted in the US that examined trial‐decision pathways to elucidate challenges to oncology trial recruitment revealed that in 56% of cases it was due to lack of available trials, 22% did not meet eligibility criteria, 15% of those who were eligible did not enroll, ultimately leading to only 8% of cancer patients participating in clinical trials.^5^

There is rich literature discussing the challenges for clinical trial recruitment, which can be trial specific but overall can be broadly classified into categories such as issues related to trial design (e.g., eligibility criteria),^6^, ^7^ accessibility (e.g., proximity to trial site),^8^, ^9^, ^10^ patient attitude (e.g., willingness to participate),^11^, ^12^ physician attitude (e.g., physician's preferences for treatment),^2^, ^11^ demographics (e.g., elderly with comorbidities),^13^, ^14^, ^15^ socioeconomic disparities (e.g., ethnic minorities and low income populations),^16^, ^17^, ^18^ and trial related factors (e.g., too many trials competing for a limited patient pool, poor trial designs with control drugs being not standard of care).^11^, ^19^, ^20^, ^21^ Based on these identified challenges, some strategies have been proposed to increase patient participation in cancer trials through (1) resolving structural barriers by increasing accessibility to trial sites, e.g., selecting and resourcing community‐based medical centers or clinics as trial sites,^11^, ^22^, ^23^, ^24^ (2) changing trial design elements such as broadening eligibility criteria, e.g., the ASCO/Friends of Cancer Research/FDA Modernizing Eligibility Criteria Project and Working Groups,^7^, ^25^, ^26^, ^27^, ^28^ and (3) encouraging under‐represented populations (e.g., ethnic minorities, socioeconomically disadvantaged populations, elderly) to participate in cancer trials by reaching out through multiple channels and by providing support to address specific needs within each population, e.g., support for transportation to trial site.^11^, ^17^, ^18^, ^29^

In this paper we further elaborate on the challenges for patient participation in oncology trials from physician, patient, healthcare system, and some trial‐related perspectives and present strategies such as use of digital healthcare technologies, real‐world data and real‐world evidence, decentralized clinical trials, pragmatic trial designs, and supportive services to increase patient participation.

## 2 CHALLENGES FOR PATIENT PARTICIPATION

The challenges to enroll enough cancer patients into clinical trials can vary by the type of cancer trials (e.g., trial enrolling patients with rare cancer), selected sites, demographic and socioeconomic factors, and trial related issues. Table 1 presents five different categories of challenges for patient recruitment from trial design and conduct, patient and physician perspectives, healthcare system, trial related issues, and other considerations. Challenges relating to trial design and conduct have been discussed extensively.^5^, ^19^, ^30^

**TABLE 1:** Challenges and strategies to increase patient participation in cancer trials

Challenges	Strategies
Trial design and conduct	
Restrictive eligibility criteria Geographical location of trial sites Need for frequent in‐person visits A limited number of pre‐defined methods for outcome assessment	Pragmatic trial designs to (1) define less restrictive eligibility criteria, (2) expand recruitment of patients from broader trial sites, (3) deliver treatment in usual care settings, (4) schedule follow‐ ups that are convenient to patients and investigators, and (5) outcomes assessed as in routine medical practice Use of real‐world data and real‐world evidence Use of digital healthcare technologies to improve patient recruitment and retention, data collection, and real‐time analytics
Patient perspective	
Patient awareness, attitude, and willingness Lack of access to trial sites Lack of supportive services (e.g., transportation, financial assistance, language translation) Lack of trust in the healthcare system	Communication, education, and transparency: appropriate promotion and propagation of cancer trials in culturally acceptable manner in community‐based settings Supportive services: (1) financial support, (2) transportation assistance, and (3) home visits Use of decentralized clinical trials to reduce participant burden and potentially increase accrual and retention of a more diverse population
Community‐based physician perspective	
Insufficient engagement Lack of information about trials Inadequate funding, resource, and comprehensive specialties Complexity of study protocols	Communicating planned trials to oncologists Educational program to improve the awareness of cancer trials and their benefits to patients Extra funding to support trial activities Connecting community‐based oncologists with those in academic centers conducting trials
Healthcare system perspective	
Insufficient resource to support clinical trials Lack of prioritization and integration of oncology trial options for patients	Providing more resource to support clinical research Building a diverse workforce to improve patient engagement and trust Leveraging large clinical databases to identify patients, especially for rare cancers
Other considerations	
Insufficient connection and partnership among physician net‐ works, medical societies, and patient advocacy organizations Inflexibility of outcome assessment and its extensive documentation Medical licenses for physicians issued by individual states that can limit the ability of trial physicians to conduct virtual visit across state lines	Establishing regional or national networks among relevant organizations (e.g., SWOG) to increase partnership for patient participation Flexible tools for outcome assessment with simplified documentation and reimbursement procedures A national license recognized in all 50 states of the United States to reduce the amount of paper work and facilitate more virtual visits to be performed for screening and study visits

### 2.1 Lack of accessibility to trials

From patient perspective, one of the major challenges is lack of accessibility to trials due to remote geographical locations (e.g., rural areas) and socioeconomic status (e.g., lack of insurance).^31^ Studies have found that patients with cancers such as metastatic prostate and colorectal cancers need to drive more than an hour each way to get to a clinical trial site and the proportion of patients driving more than an hour each way is higher in the central United States than in the coastal regions.^19^, ^32^ Selection of cancer trial sites has a profound impact not only on patient accessibility to cancer trials, but also on experience, knowledge, preference, and recommendations of treating physicians in local communities.^2^, ^33^, ^34^ The FDA recently issued a draft guidance providing recommendations for enrolling a diverse patient population into clinical trials.^35^

Willingness of travel long distances to trial sites depends on a number of factors such as availability of transportation and financial and sociocultural considerations and usually differs among different ethnic groups and geographic regions.^12^, ^36^, ^37^, ^38^

### 2.2 Awareness, attitude, and willingness

Patient's awareness of, attitude and willingness to participate have a major impact. For example, patients may be less likely to ask about trial options, be fearful of being randomized to the “placebo” arm or of experiencing unknown toxicities that are associated with experimental drugs.^39^, ^40^ Some others may not participate due to concerns of indirect costs such as travel and loss of work hours^19^ or lack of special assistance such as transportation to a trial site, language translation for those who do not speak the language used in their participating trial sites, such as English in US sites, lack of proper education to understand clinical contents, and cultural factors such as lack of trust in the healthcare system.^5^, ^24^

### 2.3 Physician‐related challenges

The common challenges for physicians in community‐based healthcare facilities include in‐ adequate engagement and funding, lack of comprehensive specialties, and system‐based challenges.^10^, ^19^ Studies have found that more than half of surveyed oncologists who were involved in cancer trials perceived that lack of information about trials, insufficient resources (e.g., too much paperwork, no research personnel to enroll participants), and complexity of protocols are the top three reasons for low accrual to oncology trials.^41^, ^42^

## 3 STRATEGIES TO INCREASE PATIENT PARTICIPATION

Based on the above discussed challenges, we discuss some strategies to increase patient participation in cancer trials, especially those enrolling patients with rare cancers (Table 1).

### 3.1 Pragmatic trial design and conduct

A classical randomized controlled trial (RCT) enrolls patients who meet a predefined set of (usually quite restrictive) eligibility criteria and consequently its results are often not generalizable to the real‐world medical practice. To address this concern, pragmatic clinical trials (PCT) have been proposed which are designed to show the real‐world effectiveness of an intervention in routine medical practice.^43^, ^44^, ^45^, ^46^ PCTs differ from RCTs in at least one of nine dimensions, the so‐called PRECIS‐2 domains^47^ and can be used to increase patient participation in cancer trials, especially for under‐represented populations. Specifically, a PCT can (1) use a set of (relatively less restrictive) eligibility criteria by including those who are most likely to use the intervention in usual care setting (e.g., the elderly, people with comorbidities), (2) expand recruitment of patients in usual care settings (e.g., small clinics) who otherwise would not have a chance to participate, (3) deliver the intervention without an additional burden to patients (e.g., visits, long stay), healthcare staff, and resource, (4) make follow‐up schedules convenient to participants and investigators and use digital health tools during follow‐up, and (5) choose primary outcomes that are important to patients, healthcare providers, and policymakers and that can easily be assessed using standard of care.^47^, ^48^, ^49^, ^50^ Note that relaxing patient eligibility criteria in a PCT will boost the patient pool and hence help recruit more patients, however it will presumably increase heterogeneity of trial participants, which may cause a sample size increase in order to achieve desired statistical power. A PCT design should take into consideration the balance of representativeness of the target population (via eligibility criteria), feasibility of trial conduct, and generalizability of trial results.^51^, ^52^ A recent simulation study using real‐world data on different eligibility criteria in clinical trials found that many common criteria, including exclusions based on several laboratory values, had minimal effect of trial estimates and conclusions, thus, not adversely impact study power.^7^

### 3.2 Real‐world data and real‐world evidence

Real‐world data (RWD) are “data relating to patient health status and/or the delivery of health care routinely collected from a variety of sources” and real‐world evidence (RWE) is “the clinical evidence about the usage and potential benefits or risks of a medical product derived from analysis of RWD.”^53^ RWD and RWE have been used in the design, analysis, and interpretation of clinical trials and in regulatory decision‐making.^54^ The use of RWD and RWE can help increase patient participation in cancer trials, especially for patients with rare cancers. For example, patient registry data with rare cancers can be used to provide the distribution of patients across different demographic factors (e.g., gender, age, ethnicity) and geographic locations (e.g., by zip code), which can help set eligibility criteria, plan patient enrollment, choose trial sites, and target recruitment efforts.^55^ Furthermore, leveraging large clinical datasets can inform patient identification for trial participation, especially in case of rare cancers. An example of such an effort is the VA Precision Oncology Program, which links patients to available clinical trials through an organized effort of a healthcare system.^23^

Some RWD may contain multiomics data (e.g., genomics, proteomics, metaboliomics, etc.), pharmacokinetics and pharmacodynamics (PK/PD) results, and observed toxicities, which can be used to help precisely locate or identify patients who would most likely benefit from a treatment, especially a targeted therapy (e.g., cancer patients carrying certain somatic mutations), or experience a harm if a particular treatment or its high dose is given to patients with certain characteristics.^56^, ^57^ In addition, some RWD sources (e.g., electronic health records) may contain a variety of alternative treatment strategies that can be considered as a standard of care to which the new therapy can be compared. Such a comparison results in treatment effectiveness because it accommodates what is available in medical practice, which represents the best interest of and hence can be more attractive to cancer patients.

### 3.3 Digital technologies

Use of digital healthcare technologies (e.g., telehealth, telemedicine, mobile health, health information technologies, and wearable devices) in trial conduct provides a great opportunity to make clinical research more inclusive, accessible, and efficient.^57^, ^58^ Current public health challenges posed by the COVID‐19 pandemic have increased the use of telemedicine in everyday oncology practice. Its implementation and wider uptake in the conduct of cancer trials can improve patient participation. Digital health expands partnership with physician networks, medical societies, and patient advocacy organizations, making clinical trials more accessible to diverse populations of cancer patients. In addition, modern digital technologies and artificial intelligence are increasingly used to enhance patient recruitment and retention. For example, block chain technology (a decentralized, distributed ledger that records the provenance of a digital asset that can be securely shared among the nodes of a computer network) uses a master smart contract for auto‐matching of potential patients based on eligibility criteria and multiple‐trial‐based smart contracts for engagement, recruitment, and retention of patients and trial management. Specifically, patient inclusion and exclusion criteria can be input into the master smart contract that uses auto‐matching functions to select potential patients based on their healthcare records (e.g., demographics, diagnosis, treatment, etc.) that are stored in trial site secure databases. Selected patients will be notified by the master smart contract for availability of clinical trials and for patient authorization to allow the eligible trial sponsor to access their electronic health records to double check recruitment criteria. Therefore, using a blockchain system, patients opt‐in and wait for notification of matched potential eligible trials to participate.^59^, ^60^, ^61^

More broadly, digital health technologies can be used for:

- *Patient recruitment*: Digital advertisements through social media such as smartphone apps that are compliant with relevant regulations and policies can be used to reach out to cancer patients. A smart contract, a self‐executing protocol running on blockchain to regulate information transactions, can (1) automatically match potential patients based on trial eligibility criteria, (2) ask matched patients for consent to join the trial, and (3) generate a specific trial contract for each trial that is accessible only to the participants.
- *Patient engagement*: After signing the consent form, patients can have access to trial materials, receive training, send messages to sponsor or investigators, and input health information. Investigators and trial participants can communicate virtually about treatments, patient‐reported outcomes, outcome assessment, adverse experiences, and any other trial‐related concerns and activities.^30^
- *Therapeutic intervention and data collection*: Telemedicine can be used by physicians to provide instructions to trial participants on home‐based delivery of investigational products and on scheduled study visits. Patient experience (e.g., adverse events) and outcomes (including patient‐reported outcomes or PROs) can be assessed remotely and collected in a timely manner.^62^
- *Real‐time analytics*: With trial data being entered and analytic methods being built into the digital system, trial data can be analyzed in real time for monitoring the quality of the trial (including missing values), performing pre‐planned analyses, and combining data from different sources for integrated summaries of efficacy and safety.^30^

The SWOG (South‐Western Oncology Group) provides a good example of using digital engagement in improving clinical infrastructure in (1) expanding the SWOG community, (2) increasing public awareness of cancer trials, and (3) remote informed consent.^63^

### 3.4 Decentralized clinical trials

The FDA recently proposed decentralized clinical trials (DCTs) which allow some trial activities to be performed at non‐trial sites and even participant's homes.^64^ DCTs have the advantages over traditional RCTs in terms of convenience, travel, and participation inclusiveness and therefore appeal to a wide range of participants by reducing financial burden and commitments required for participation.^65^ DCTs can also improve participant inclusiveness and hence facilitate the participation of under‐represented population.^66^ Digital technologies such as telemedicine and wearable devices are commonly used for diagnosis and monitoring. Some challenges in the design and conduct of DCTs may include patient heterogeneity (see “Pragmatic clinical trials” section for more discussion), appropriate use of digital technologies at home (more training is needed for participants), investigator's oversight on patient safety, and more complex infrastructure for trial conduct^64^, ^65^

### 3.5 Awareness, communication, and education

From patient perspective, appropriate advertisement and propagation of cancer trials in a culturally acceptable manner in community‐based settings, e.g., advertisement through multi‐channels in different languages, may engage more patients to participate. Educational and recruitment materials, informed consent documents, presentation of early study results, etc., can be designed in a culturally and linguistically adaptive way that promote clear understanding of the research questions, study design, participant protections, and potential individual and community benefits^67^ and these materials can be placed in community‐based medical centers or clinics or even online for easy access. On the other hand, upon locating eligible patients using RWD, media advertising campaign such as smartphone apps, mailing flyers, and so on can be considered for propagation, communication, and educational purpose. It is important to invest and develop collaborations with community organizations to engage with the patient population before trial enrollment starts.

From physician perspectives, communicating planned clinical trials to and engaging community‐based oncologists are a way to broaden target populations and increase cancer patient enrollment. Educational programs for community‐based oncologists about the importance of cancer trials and the benefit to their patients and creating connections between community‐based oncologists and clinical trialists in academic centers can be beneficial.

### 3.6 Prioritization, information integration, and diverse workforce in healthcare system

From healthcare system perspective, the prioritization and integration of oncology trial options for patients and providing adequate resources to support clinical research are essential for the success of clinical trials. When multiple trials compete for a limited patient pool, recruitment can be prioritized based on potential benefits and risks of individual trials to trial participants (e.g., evidence from earlier studies). Frequent review of clinical trial portfolios by trial sites and sponsors to potentially close studies that are underperforming or where the data are no longer supportive of continued enrollment optimizes trial opportunities available for patients. Healthcare systems should also focus on building a diverse workforce which improves patient engagement and builds trust.^68^, ^69^

### 3.7 Supportive care services

Patients may have special needs to participate in cancer trials. Those with low income or who are socioeconomically disadvantaged may need financial support for their participation (e.g., travel expenses, time away from work for them and their caregivers, medical insurance coverage, lodging).^37^, ^38^ For example, a study showed that the implementation of the cancer care equity program resulted in an increase in cancer trial participation, which highlights the need to address the financial burden associated with trial participation.^36^ Travel assistance may be provided to cancer patients who cannot afford transportation services (e.g., bus/train or taxi) or for whom public transportation is not readily available. For patients with physical challenges (e.g., the elderly, the handicapped), medical or special transportation services may be provided or investigators can schedule home visits to allow patients to participate in the trial.^12^

### 3.8 Other considerations

Clinical trials require patients to undergo multiple evaluations including specialized blood tests and radiologic imaging, which is usually available at bigger healthcare centers. Out‐ come assessments using sophisticated tools and the need for extensive documentation of laboratory and radiology facilities can present a barrier to trial participation. Consideration should be given to type and frequency of testing, weighing it against need to meet research objectives, ensure patient safety and allow for patient convenience. For example, patients should be allowed to go local laboratories for some testing with mechanisms set up for financial coverage of research testing at these locations. The amount of documentation required to accept radiologic and laboratory data from various sites should be reconsidered with standard certifications being sufficient for trial related testing to be performed and submitted. Reimbursement for research related tests also limits the ability of patients to obtain testing at sites other than those pre‐certified in the research contracts. Going from state specific physician license to a national license will improve ability of patients to receive care across state lines and enroll more easily in out of state trials.

## 4 CONCLUDING REMARKS

Low patient participation in clinical trials, especially oncology trials, continues to be a challenging issue, which hampers development of innovative anticancer therapies. Some strategies are proposed to overcome the discussed challenges, and include the use of digital healthcare technologies for patient accrual, virtual visits, and outcome assessment, RWD and RWE for identifying, locating, and enrolling cancer patients, pragmatic trial design to include more patients and make trial conduct closer to routine medical practice, decentralized clinical trials to reduce patient burden and potential increase accrual and retention of a more diverse trial population, and supportive services to help patients access trial sites. Finally, communication, education, and transparency are essential for patients and physicians to understand the importance of cancer trials and their role in improving health outcomes.

## AUTHOR CONTRIBUTION

All authors have contributed to the writing and approval of the manuscript.

## CONFLICT OF INTEREST

J Chen: Employee of Overland Pharmaceuticals. Y Lu: Serving as a member of data monitoring committees for Nektar, Gilead, Genentech, and data safety committee for the Emergent BioSolutions. S Kummar: Consultant/Advisory board: Boehringer Ingelheim, Springworks Therapeutics, Bayer, Genome & Company, HarbourBiomed, Oxford Biotherapeutics, Mundibiopharma, Gilead, EcoR1, Mirati, Bayer; PathomIQ (co‐founder), Cadila Pharmaceuticals (scientific advisor‐spouse), and Arxeon (co‐founder‐spouse).

## INSTITUTIONAL REVIEW BOARD (IRB) OR ETHICS COMMITTEE APPROVAL

N/A; this is a review of already published data and does not meet criteria for seeking IRB or Ethics Committee approval.

## STUDIES INVOLVING HUMAN PARTICIPANTS OR PATIENT MATERIAL (TISSUE/BLOOD SAMPLES)

N/A.

## Data Availability

Not Applicable.
